# Hierarchical Co_3_O_4_ Nano‐Micro Arrays Featuring Superior Activity as Cathode in a Flexible and Rechargeable Zinc–Air Battery

**DOI:** 10.1002/advs.201802243

**Published:** 2019-03-26

**Authors:** Yaotang Zhong, Zhenghui Pan, Xianshu Wang, Jie Yang, Yongcai Qiu, Shuyuan Xu, Yitong Lu, Qiming Huang, Weishan Li

**Affiliations:** ^1^ School of Chemistry and Environment South China Normal University Guangzhou 510006 China; ^2^ Department of Materials Science and Engineering National University of Singapore Singapore 117574 Singapore; ^3^ School of Environment & Energy South China University of Technology Guangzhou 510006 Guangdong China; ^4^ Engineering Research Center of MTEES (Ministry of Education) Research Center of BMET (Guangdong Province) and Key Laboratory of ETESPG (GHEI) South China Normal University Guangzhou 510006 China

**Keywords:** cathodes, Co_3_O_4;_ nano‐micro arrays, superior activities, zinc–air batteries

## Abstract

All‐solid‐state zinc–air batteries are characterized as low cost and have high energy density, providing wearable devices with an ideal power source. However, the sluggish oxygen reduction and evolution reactions in air cathodes are obstacles to its flexible and rechargeable application. Herein, a strategy called MOF‐on‐MOF (MOF, metal‐organic framework) is presented for the structural design of air cathodes, which creatively develops an efficient oxygen catalyst comprising hierarchical Co_3_O_4_ nanoparticles anchored in nitrogen‐doped carbon nano‐micro arrays on flexible carbon cloth (Co_3_O_4_@N‐CNMAs/CC). This hierarchical and free‐standing structure design guarantees high catalyst loading on air cathodes with multiple electrocatalytic activity sites, undoubtedly boosting reaction kinetics, and energy density of an all‐solid‐state zinc–air battery. The integrated Co_3_O_4_@N‐CNMAs/CC cathode in an all‐solid‐state zinc–air battery exhibits a high open circuit potential of 1.461 V, a high capacity of 815 mAh g^−1^ Zn at 1 mA cm^−2^, a high energy density of 1010 Wh kg^−1^ Zn, excellent cycling stability as well as outstanding mechanical flexibility, significantly outperforming the Pt/C‐based cathode. This work opens a new door for the practical applications of rechargeable zinc–air batteries in wearable electronic devices.

## 1 Introduction

The increasing demands for “green technology” have gained extensive interests in efficient, inexpensive, stable, renewable, cleaner energy conversion, and storage devices. Rechargeable zinc–air battery (ZAB), regarded as one of most promising eco‐friendly “green technology,” has been valued most by its high theoretical specific energy density of 1086 Wh kg^−1^ Zn, low cost, and high safety.^1^, ^2^, ^3^, ^4^, ^5^ However, the previously reported ZABs have been generally designed as unsealed aqueous devices with gas flow, which are complicated, unsafe, and high‐cost.^6^, ^7^ Comparatively, with no need for sealing, the flexible all‐solid‐state ZABs have attracted much attention due to the advantages of higher practicability, safety, and lower cost, showing a brighter future for various applications.^8^

Unfortunately, the traditional flexible all‐solid‐state ZABs are still suffering from relatively low energy density owing to the sluggish oxygen evolution reaction (OER) and oxygen reduction reaction (ORR), especially with the insufficient catalytic active materials on air cathodes and the “dead mass” caused by the polymeric binders/conductive additives.^9^, ^10^, ^11^, ^12^ Although much effort has been made to improve the OER and ORR activity of air cathodes by designing various nanomaterials, these researches totally ignore the significance of the catalytic active materials loading on air cathodes toward energy density promotion.^13^, ^14^, ^15^, ^16^, ^17^ To address these issues, we propose a novel synthetic strategy for cathode fabrication, called MOF‐on‐MOF (MOF, metal‐organic framework). The 3D and 2D cobalt‐based MOFs (Co‐MOF) with different crystal structures, morphologies, and particle sizes, were grown on carbon cloth (CC), simply by changing solvent only. Then, a binder‐free cathode, ultimately hierarchical Co_3_O_4_ nanoparticles anchored in nitrogen‐doped carbon nano‐micro arrays (Co_3_O_4_@N‐CNMAs), was fabricated by a facile carbonization‐oxidation process, which features high specific surface area and active material loading. The 2D Co‐MOF directly grown on carbon cloth as the support provides ample space for 3D Co‐MOF growth, resulting in a hierarchical 3D‐on‐2D MOF architecture with more catalytic active sites exposed to the electrolyte compared with only 3D or 2D MOF. Notably, this hierarchical MOF‐on‐MOF structure is facilely assembled on CC without using any structure‐directed and binder agent, providing the cathode with higher active material and more catalytic active sites, compared with the single MOF‐derivative structures or other MOF‐on‐MOF core–shell structures reported previously.^18^, ^19^ Besides, these N‐CNMAs derived from Co‐MOF can well prevent the aggregation of Co_3_O_4_ nanoparticles and further promote the electron transfer during the electrocatalytic process. Therefore, such a synergetic effect stimulates an excellent electrocatalytic activity and stability toward both OER and ORR. As a proof‐of‐concept application, the hierarchical Co_3_O_4_@N‐CNMAs/CC electrode is directly applied as an air cathode without any binder in a flexible and rechargeable solid‐state ZAB, which exhibits a high open circuit potential of 1.461 V, a high capacity of 815 mAh g^−1^ Zn at the current density of 1 mA cm^−2^, a high energy density of 1010 Wh kg^−1^ Zn, excellent cycling stability as well as outstanding flexibility.

## 2 Results and Discussion

**Figure 1:**
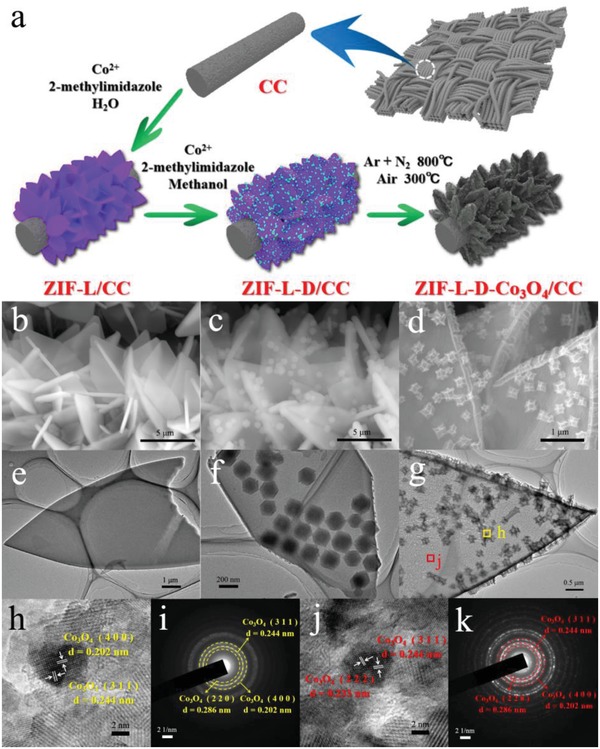
a) Schematic illustration of the formation process of ZIF‐L‐D‐Co_3_O_4_/CC; b–d) SEM images and e–g) TEM images of ZIF‐L/CC, ZIF‐L‐D/CC, and ZIF‐L‐D‐Co_3_O_4_/CC; h,i) HRTEM and SAED images of ZIF‐D‐Co_3_O_4_ and j,k) ZIF‐L‐Co_3_O_4_.

The formation process of the hierarchical Co_3_O_4_ based 3D‐on‐2D nano‐micro arrays is schematically illustrated in **Figure** **1**a, and the microstructure of the as‐prepared samples in each step was characterized by scanning electron microscopy (SEM) and transmission electron microscopy (TEM). At the beginning, 2D leaflike microarrays (denoted as ZIF‐L) with the sizes about 5 µm are uniformly grown on carbon cloth (ZIF‐L/CC) through a facile solution method (Figure 1b,e). And interestingly, under the excessive amount of 2‐methylimidazole, the size of ZIF‐L is continually increasing as the amount of Co(NO_3_)_2_·6H_2_O increases, as shown in Figure S1 in the Supporting Information, leading to a larger 2D leaf morphology.^20^, ^21^ In addition, to clearly track the growth trajectories, the growth process of ZIF‐L/CC was intensively recorded by SEM (Figure S2, Supporting Information) and X‐ray diffraction (XRD) (Figure S3, Supporting Information). Then, as presented in Figure 1c,f, 3D‐on‐2D MOF nano‐micro arrays were obtained simply by changing the water to methanol solution. The small 3D dodecahedral particles (ZIF‐D, *d* ≈200 nm) are uniformly anchored on 2D ZIF‐L microarrays. Moreover, as time goes on, the numbers of anchored ZIF‐D on ZIF‐L are gradually increased (Figure S4, Supporting Information). Finally, the 3D‐on‐2D MOF precursor (denoted as ZIF‐L‐D) was processed by means of a facile carbonization‐oxidation method, during which the organic ligands are transformed into porous nitrogen‐doped (N‐doped) carbon, and Co ions are transformed into Co_3_O_4_ nanoparticles. As can be seen from Figure 1d,g, the well‐formed Co_3_O_4_ nanoparticles anchored in nitrogen‐doped carbon nano‐micro arrays (denoted as ZIF‐L‐D‐Co_3_O_4_) possesses an angular and rough morphology, and the Co_3_O_4_ nanoparticles are uniformly embedded in the 3D and 2D carbon matrixes (denoted as ZIF‐D‐Co_3_O_4_ and ZIF‐L‐Co_3_O_4_, respectively) (Figure S5, Supporting Information). The high‐resolution TEM (HRTEM) and selected area electron diffraction (SAED) images (Figure 1h–k) indicate that the diameters of hierarchical Co_3_O_4_ nanoparticles are about 5 nm both in ZIF‐D‐Co_3_O_4_ and ZIF‐L‐Co_3_O_4_. The as‐formed Co_3_O_4_ nanoparticles can be exactly confirmed by XRD pattern, and all diffraction are well consistent with the Co_3_O_4_ (JCPDS # 42‐1467) (Figure S6a, Supporting Information). Meanwhile, ZIF‐L‐D‐Co_3_O_4_ was further studied by X‐ray photoelectron spectroscopy (XPS). The obtained results are shown in Figure S6b and Table S1 in the Supporting Information, indicating that the ZIF‐L‐D‐Co_3_O_4_/CC comprises Co, C, N, and O without other impurities. In addition, the oxidation states of Co and N were analyzed, as demonstrated in Figure S6c,d in the Supporting Information. Two major peaks at 780.5 and 795.6 eV in Co 2p spectrum, corresponding to Co 2p_3/2_ and Co 2p_1/2_ respectively, indicate the presence of Co_3_O_4_.^22^ And the N 1s XPS spectrum can be deconvoluted into four peaks, corresponding to pyridinic‐N, graphitic‐N, oxidized‐N, and Co–N*_x_*, which suggests that the nitrogen in ZIF‐L‐D‐Co_3_O_4_/CC is present in the forms of N‐doped species.^9^, ^23^

**Figure 2:**
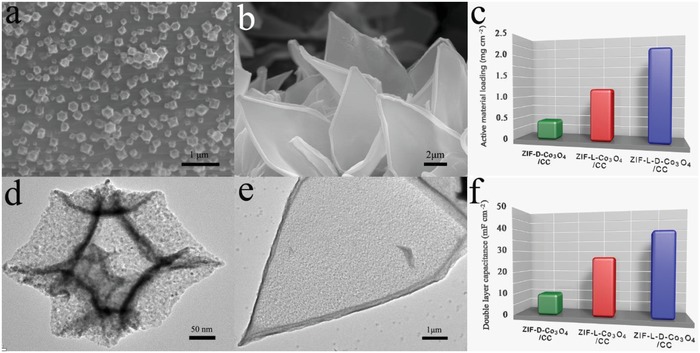
a,b) SEM images and d,e) TEM images of ZIF‐D‐Co_3_O_4_/CC and ZIF‐L‐Co_3_O_4_/CC; The comparison of c) active material loading and f) double layer capacitance of ZIF‐D‐Co_3_O_4_/CC, ZIF‐L‐Co_3_O_4_/CC, and ZIF‐L‐D‐Co_3_O_4_/CC.

During the formation process, it became obvious that the specific surface area of the ZIF‐L‐D‐Co_3_O_4_/CC was noticeably higher than their precursors from Brunner–Emmet–Teller (BET) results (Figure S7, Supporting Information), indicating that the higher electrochemical active surface area. For comparison, the ZIF‐D‐Co_3_O_4_ and ZIF‐L‐Co_3_O_4_ were separately decorated on CC (denoted as ZIF‐D‐Co_3_O_4_/CC and ZIF‐L‐Co_3_O_4_/CC). As shown in **Figure** **2**a,b, ZIF‐D‐Co_3_O_4_ and ZIF‐L‐Co_3_O_4_ are compactly grown on CC, with an even distribution of Co_3_O_4_ (Figure 2d,e). The electrochemical active surface area (Figure 2f and Figure S8: Supporting Information) was evaluated by the double‐layer capacitance (*C*_dl_), affording 9.98, 26.65, and 38.56 mF cm^−2^ for ZIF‐D‐Co_3_O_4_/CC, ZIF‐L‐Co_3_O_4_/CC, and ZIF‐L‐D‐Co_3_O_4_/CC, respectively. Meanwhile, the mass loading of ZIF‐Co_3_O_4_ on CC (Figure 2c) was measured by weighing, 0.4 mg cm^−2^ for ZIF‐D‐Co_3_O_4_/CC, 1.2 mg cm^−2^ for ZIF‐L‐Co_3_O_4_/CC, and 2.1 mg cm^−2^ for ZIF‐L‐D‐Co_3_O_4_/CC. Further, the mass loading of Co_3_O_4_ and the ratio of Co_3_O_4_ on 3D to that on 2D in the cathode were obtained by etching ZIF‐L‐D‐Co_3_O_4_/CC and ZIF‐L‐Co_3_O_4_/CC with dilute HCl to remove Co_3_O_4_ and comparing the mass losses before and after etching. As shown in Table S2 in the Supporting Information, the mass loading of Co_3_O_4_ in ZIF‐L‐D‐Co_3_O_4_/CC is about 1.82 mg cm^−2^ and the ratio of Co_3_O_4_ on 3D to that on 2D is about 4:5. Correspondingly, the mass loading of Co_3_O_4_ in ZIF‐D‐Co_3_O_4_/CC and ZIF‐L‐Co_3_O_4_/CC are 0.31 and 1.01 mg cm^−2^, respectively. The higher electrochemical active surface area and active material loading greatly guarantee that the ZIF‐L‐D‐Co_3_O_4_/CC has multiple electrocatalytic activity sites compared with the other two samples.^24^

**Figure 3:**
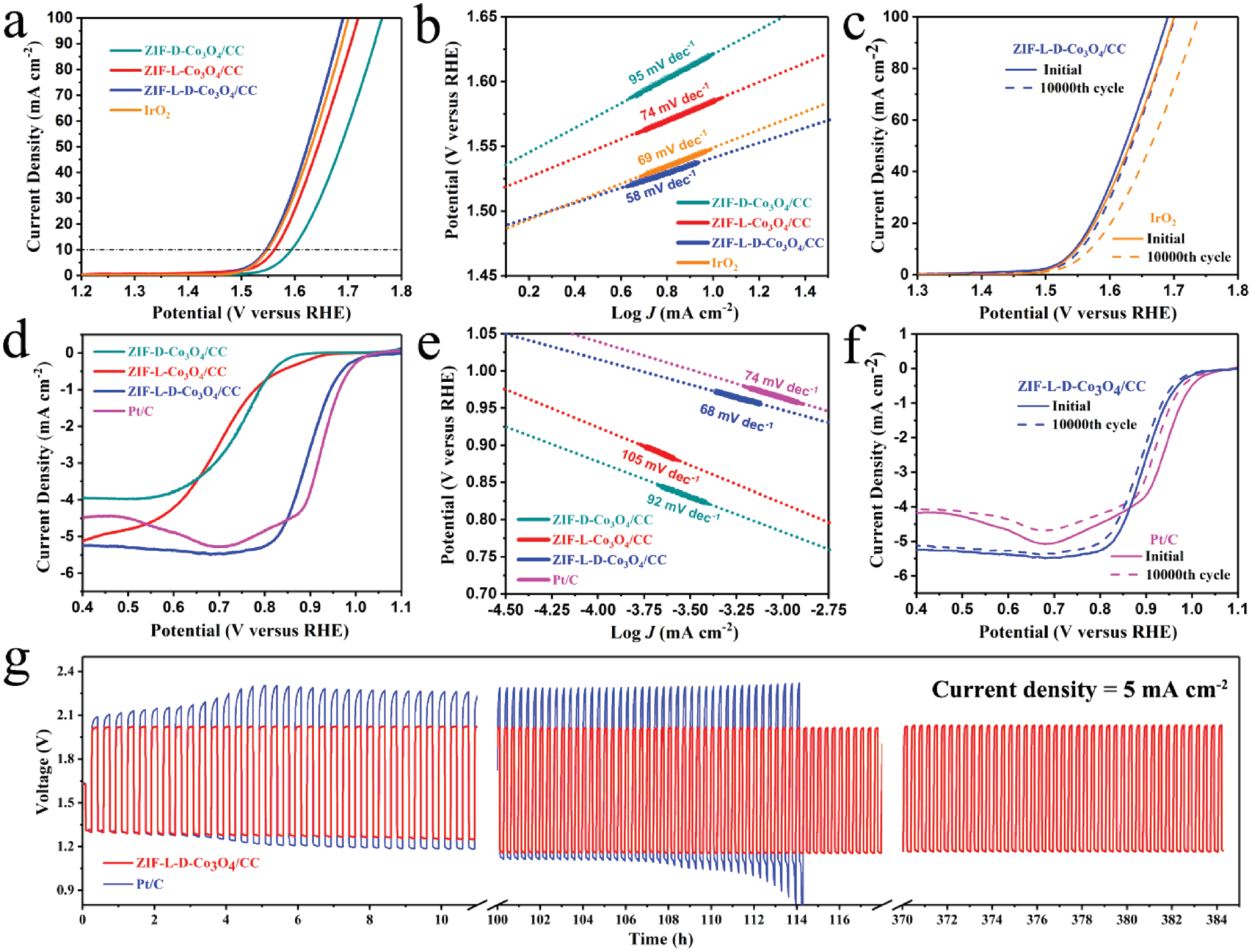
a) Polarization curves, b) Tafel plots, c) polarization curves before and after 10 000 potential sweeps between 1.4 and 1.6 V at 50 mV s^−1^ for OER test; d) Polarization curves, e) Tafel plots, f) polarization curves before and after 10 000 potential sweeps between 0.8 and 1.0 V at 50 mV s^−1^ for ORR test; g) Comparison of stability test between ZIF‐L‐D‐Co_3_O_4_/CC and Pt/C as the air cathode in aqueous ZABs.

To evaluate the electrocatalytic performance of ZIF‐L‐D‐Co_3_O_4_/CC, the OER and ORR were first investigated in a three‐electrode cell using 1 m potassium hydroxide (KOH) solution saturated by oxygen. For comparison, the commercial IrO_2_ and Pt/C, and the resulting ZIF‐D‐Co_3_O_4_/CC and ZIF‐L‐Co_3_O_4_/CC were tested as well. It should be noted that all the samples were directly attached to a glassy carbon electrode and were then carefully studied on a rotating disk electrode (see Figure S9 in the Supporting Information for details). For OER, as shown in **Figure** **3**a, ZIF‐L‐D‐Co_3_O_4_/CC requires a lower overpotential of 310 mV (vs standard potential of O_2_ evolution) to achieve the current density of 10 mA cm^−2^, compared to those of IrO_2_ (320 mV), ZIF‐L‐Co_3_O_4_/CC (340 mV), and ZIF‐D‐Co_3_O_4_/CC (370 mV). Moreover, ZIF‐L‐D‐Co_3_O_4_/CC also displays the lowest Tafel slope (58 mV dec^−1^), remarkably lower than IrO_2_ (69 mV dec^−1^), ZIF‐L‐Co_3_O_4_/CC (74 mV dec^−1^), and ZIF‐D‐Co_3_O_4_/CC (95 mV dec^−1^) (Figure 3b), indicative of the better activity of ZIF‐L‐D‐Co_3_O_4_/CC than ZIF‐D‐Co_3_O_4_/CC and ZIF‐L‐Co_3_O_4_/CC toward OER. Most importantly, ZIF‐L‐D‐Co_3_O_4_/CC exhibits a superior OER stability (Δ*E* ≈10 mV) than IrO_2_ (Δ*E* ≈ 30 mV) after 10 000 cycles (Figure 3c). Such an excellent stability can be strongly confirmed by the chronoamperometric response at a constant current density of 10 mA cm^−2^ and chronopotentiometric responses at a constant overpotential of 320 mV (vs standard potential of O_2_ evolution) (Figure S10a, Supporting Information). The outstanding OER performances of ZIF‐L‐D‐Co_3_O_4_/CC can be attributed to the unique hierarchical 3D‐on‐2D structure with multiple electrocatalytic activity sites and free‐standing architecture, which undoubtedly boosts reaction kinetics and mass transport of ionic species such as OH^−^, O_2_^2−^, and O^2−^.^25^, ^26^ In addition, owing to the electron‐withdrawing nature of N‐doping in the carbon, positively charged Co–N*_x_* and C–N species facilitate the adsorption of OH^−^, benefiting the OER kinetics.^7^, ^22^, ^27^, ^28^, ^29^, ^30^

Likewise, the ZIF‐L‐D‐Co_3_O_4_/CC presents substantial improvement in ORR properties, compared with the ZIF‐L‐Co_3_O_4_/CC and ZIF‐D‐Co_3_O_4_/CC, as shown in Figure 3d. Strikingly, the ZIF‐L‐D‐Co_3_O_4_/CC exhibits an onset potential of 0.97 V with a half‐wave potential of 0.90 V, which is better than those of ZIF‐L‐Co_3_O_4_/CC (an onset potential of 0.91 V with a half‐wave potential of 0.70 V) and ZIF‐D‐Co_3_O_4_/CC (an onset potential of 0.84 V with a half‐wave potential of 0.74 V), and very close to those of the state‐of‐the‐art Pt/C (an onset potential of 1.00 V with a half‐wave potential of 0.94 V). More interestingly, as shown in Figure 3e, the estimated Tafel slope of ZIF‐L‐D‐Co_3_O_4_/CC (68 mV dec^−1^) is even lower than that of Pt/C (74 mV dec^−1^), signaling its favorable ORR kinetics. Apart from the high activity, the ZIF‐L‐D‐Co_3_O_4_/CC also exhibits a considerable ORR stability after 10 000 cycles (Δ*E* ≈10 mV), which obviously outperforms that of Pt/C (Δ*E* ≈40 mV) (Figure 3f). The chronoamperometric and chronopotentiometric responses (Figure S10b, Supporting Information) simultaneously provide further evidence of such excellent ORR stability. To gain in‐depth information on the ORR mechanism, the ORR polarization curves were recorded at different rotation speeds and the corresponding Koutecky–Levich (K–L) plots were obtained (Figure S11, Supporting Information). All the K–L plots at different potentials show good linearity, indicating first‐order reaction kinetics toward dissolved oxygen, and similar electron‐transfer number (*n*) during the ORR process. Based on the average values calculated from different potentials, the *n* of ZIF‐L‐D‐Co_3_O_4_/CC is calculated to be ≈3.91, which is close to the theoretical value of Pt/C (4.0) and far larger than those of the ZIF‐D‐Co_3_O_4_/CC (3.12) and ZIF‐L‐Co_3_O_4_/CC (3.41), indicating that the ZIF‐L‐D‐Co_3_O_4_/CC exhibits better ORR performance. These results robustly demonstrate that the ZIF‐L‐D‐Co_3_O_4_/CC featuring multiple electrocatalytic activity sites can function well in ORR performance because of improved oxygen‐adsorption, efficient ion‐diffusion and electron‐transfer.^16^, ^31^, ^32^ Last but not least, the superior OER and ORR properties of the ZIF‐L‐D‐Co_3_O_4_/CC can be contributed to the close protection of N‐doped carbon on Co_3_O_4_ nanoparticles, which alleviates the aggregation of Co_3_O_4_ and suppresses its direct exposure to electrolyte, offering excellent electrical conductivity and electrochemical stability.^33^, ^34^, ^35^, ^36^ It can be noted that the improved activity of ZIF‐L‐D‐Co_3_O_4_/CC is not proportional to its mass loading of Co_3_O_4_ (1.82 mg cm^−2^), which is significantly larger than that of Co_3_O_4_ in ZIF‐D‐Co_3_O_4_/CC (0.31 mg cm^−2^) or ZIF‐L‐Co_3_O_4_/CC (1.01 mg cm^−2^). This phenomenon can be explained by the partially unexploited Co_3_O_4_ in ZIF‐L‐D‐Co_3_O_4_/CC, and it is not an issue for the practical application of ZIF‐L‐D‐Co_3_O_4_/CC as a catalyst compared to the noble platinum.

As a proof‐of‐concept application, an aqueous ZAB was designed using ZIF‐L‐D‐Co_3_O_4_/CC as the air cathode (see Figure S12 (Supporting Information) and the Experimental Section for detail). With the superior activity of ZIF‐L‐D‐Co_3_O_4_/CC as the air cathode, the as‐assembled ZIF‐L‐D‐Co_3_O_4_/CC‐based aqueous ZAB presents a higher open circuit voltage (1.660 V), a larger power density (75 mW cm^−2^), and a larger specific capacity (852 mAh g^−1^ Zn at 5 mA cm^−2^) than the Pt/C‐based ZAB (1.625 V, 63 mW cm^−2^, and 640 mAh g^−1^ Zn, respectively), as demonstrated in Figure S13 in the Supporting Information. The catalytic stability of ZIF‐L‐D‐Co_3_O_4_/CC was further evaluated under continuous galvanostatic discharging/charging at 5 mA cm^−2^ with each cycle being 10 min. Figure 3g obviously reveals that after about 384 h long‐time testing, the ZIF‐L‐D‐Co_3_O_4_/CC‐based ZAB only presents a slight performance loss with a small voltage gap between charge and discharge about 0.83 V. The images of the cycled ZIF‐L‐D‐Co_3_O_4_/CC electrode (Figure S14, Supporting Information) indicate that the hierarchical structural morphology of ZIF‐L‐D‐Co_3_O_4_ is maintained, though the size of Co_3_O_4_ nanoparticles in ZIF‐L‐D‐Co_3_O_4_/CC is reduced slightly compared to the pristine ones (Figure 1h,j). The reduced particle size can be ascribed to the inevitable mass loss of the active components under the potential for OER.^37^ These results confirm the excellent OER and ORR activities and stabilities of the ZIF‐L‐D‐Co_3_O_4_/CC. On the contrary, although the Pt/C‐based ZAB can maintain a voltage gap about 1.12 V in the first few cycles, it increased drastically after only 110 h testing, indicating poor OER and ORR stabilities. Most probably, the observed performance decay for the ZIF‐L‐D‐Co_3_O_4_/CC‐based ZAB may be due to a few small losses of active sites during long‐time cycle, while the Pt/C‐based ZAB is suffered from the detachment of Pt nanoparticles from the current collect and particle agglomeration.^37^, ^38^ Therefore, the ZIF‐L‐D‐Co_3_O_4_/CC‐based ZAB exhibits a much better cycling durability than that of state‐of‐the‐art Pt/C‐based, showing a brighter future for aqueous energy storage devices.

**Figure 4:**
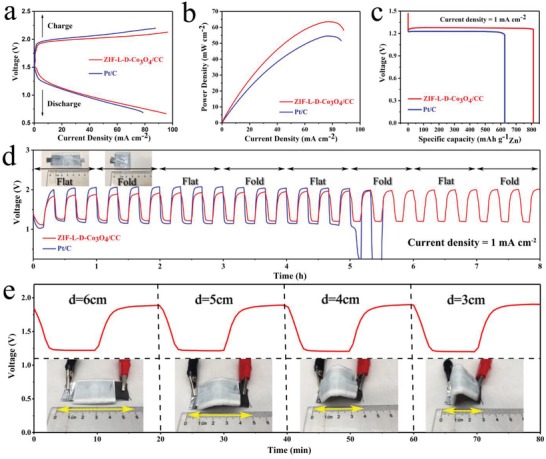
a) Discharge and charge polarization curves, b) power–current density curves, c) voltage–capacity curves of solid‐state ZABs with air cathodes of ZIF‐L‐D‐Co_3_O_4_/CC and Pt/C. d) Mechanical flexibility and stability tests of the solid‐state rechargeable ZABs under continuously mechanical altering (digital images in inset) for every three cycles. e) Discharge–charge voltage profiles of ZIF‐L‐D‐Co_3_O_4_/CC at 1 mA cm^−2^ (10 min for discharging and 10 min for charging) at the flat condition and different bending conditions.

For the applications to wearable electronic devices, we have further developed a flexible all‐solid‐state ZAB composed of a free‐standing ZIF‐L‐D‐Co_3_O_4_/CC air cathode and Zn foil anode sandwiched with alkaline poly(vinyl alcohol) (PVA) gel electrolyte (see Figure S15a (Supporting Information) and the Experimental Section for detail). As shown in Figure S15b in the Supporting Information, the assembled ZIF‐L‐D‐Co_3_O_4_/CC‐based all‐solid‐state ZAB delivers a high open circuit potential of 1.461 V. Figure S15c in the Supporting Information represents its excellent cycling stability without noticeable degradation compared with the Pt/C‐based one. Also, during the whole cycling test, the ZIF‐L‐D‐Co_3_O_4_/CC‐based ZAB always shows much lower charging voltage and higher discharging platform than the Pt/C‐based one, suggesting a higher energy efficiency. Furthermore, **Figure** **4**a displays the polarization curves of all‐solid‐state ZABs, where the ZIF‐L‐D‐Co_3_O_4_/CC exhibits a lower voltage gap between charge and discharge compared with Pt/C, indicating much better charge–discharge ability. Figure 4b presents the power–current density curves for all‐solid‐state ZABs. The maximum power density of the ZIF‐L‐D‐Co_3_O_4_/CC‐based ZAB is 65 mW cm^−2^, higher than that of the Pt/C‐based (55 mW cm^−2^). As displayed in Figure 4c, at a current density of 1 mA cm^−2^, the ZIF‐L‐D‐Co_3_O_4_/CC‐based ZAB can deliver a capacity of 815 mAh g^−1^ Zn, which is far higher than that of Pt/C‐based (625 mAh g^−1^ Zn), contributing to a high energy density of about 1010 Wh kg^−1^ Zn. As shown in Figure S16 in the Supporting Information, ZIF‐L‐D‐Co_3_O_4_/CC‐based and Pt/C‐based ZABs display similar electrochemical impedance spectra with a semicircle at high frequencies and a straight line at low frequencies, which can be well fitted by the equivalent (the inset of Figure S16 in the Supporting Information). The semicircle represents the charge transfer on the electrode/solution, while the straight line is related to the mass transfer in the solution.^39^, ^40^, ^41^ It can be found that ZIF‐L‐D‐Co_3_O_4_/CC‐based ZAB has a smaller charge transfer resistance and a larger slope than Pt/C‐based ZAB. Obviously, the unique hierarchical 3D‐on‐2D structure in ZIF‐L‐D‐Co_3_O_4_/CC provides a great number of electrocatalytic activity sites for charge transfer and paths for mass transfer, which reduce the electrochemical impedance and lead to the excellent performances of ZIF‐L‐D‐Co_3_O_4_/CC‐based ZAB,^42^, ^43^, ^44^ comparable with other all‐solid‐state ZABs reported by previous literature (Table S3, Supporting Information).^2^, ^9^, ^10^, ^35^, ^37^, ^45^, ^46^

In order to evaluate the mechanical flexibility and cycling stability of the ZIF‐L‐D‐Co_3_O_4_/CC‐based ZAB, it was carefully tested alternating between its flat (unbent, as shown in inset) and folded (bent 180°, as shown in inset) states for every three cycles, applying galvanostatic pulses for 10 min of discharge followed by 10 min of charge at 1 mA cm^−2^ (Figure 4d). During the 8 h test with severe structural changes, the ZIF‐L‐D‐Co_3_O_4_/CC‐based ZAB exhibits the stable charge (1.90 V) and discharge (1.20 V) voltages, and much less variations than the Pt/C‐based one, showing that the ZIF‐L‐D‐Co_3_O_4_/CC‐based ZAB can better buffer and alleviate the mechanical change. To further assess its flexibility and stability, the ZIF‐L‐D‐Co_3_O_4_/CC‐based ZAB was bent under different conditions to change the end‐to‐end distance. As shown in Figure 4e, under the flat and different bending conditions, the discharging and charging voltage profiles are almost the same, further confirming its excellent mechanical flexibility and cycling stability.

**Figure 5:**
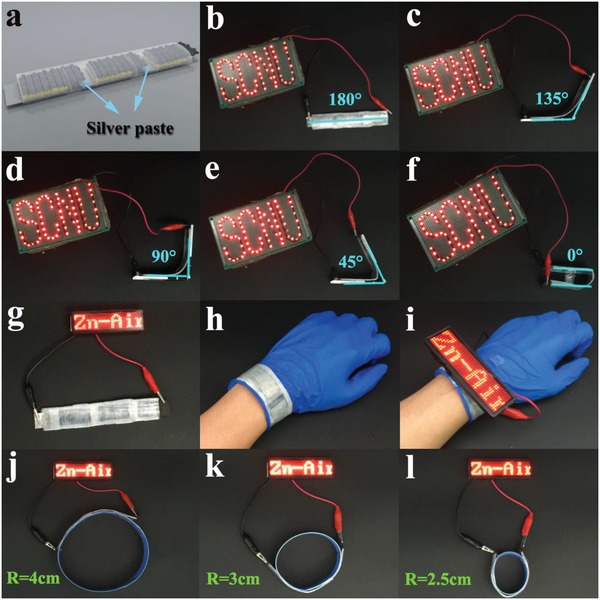
a) Schematic representation of three all‐solid‐state ZABs in series. Three‐series ZABs power 75 red LEDs with “SCNU” shape in different bending angles of b) 180°, c) 135°, d) 90°, e) 45°, and f) 0°. Photograph of g) a red LED breastpiece (3 V) activated by three‐series ZABs in flat state, h) three‐series ZABs as a wearable bracelet and i) power the breastpiece. Three‐series ZABs power a red LED breastpiece (3 V) in different bending radius of j) 4, k) 3, and l) 2.5 cm.

To practically apply the flexible all‐solid‐state ZAB to wearable electronic devices, three ZIF‐L‐D‐Co_3_O_4_/CC‐based all‐solid‐state ZABs were connected in series by silver paste (**Figure** **5**a), which can generate a high voltage of about 4.3 V and be widely applied to more electronic devices. As a demo shown in Figure 5b–f, the three‐series ZABs are capable of lighting 75 red light‐emitting diodes (LEDs, 1.6V) with “SCNU” shape under bending angles, directly emphasizing the practicability of the ZIF‐L‐D‐Co_3_O_4_/CC‐based all‐solid‐state ZAB in realistic electronic devices. Interestingly, the three‐series ZABs were also designed as a wearable bracelet, which can power a red LED breastpiece (3.0 V) on hand (Figure 5g–i). Furthermore, even under different bending radius conditions (Figure 5j–l), the wearable bracelet can still work perfectly, indicating outstanding flexibility and stability (refer to Video S1 in the Supporting Information). As demonstrated above, there is no doubt that the ZIF‐L‐D‐Co_3_O_4_/CC‐based all‐solid‐state ZAB is successfully applied to realistic wearable electronic devices, and also can push the enormous advance of next‐generation flexible energy conversion and storage devices.

## 3 Conclusion

Successfully and creatively, we have developed a facile synthetic strategy called MOF‐on‐MOF for scalable preparation of the ZIF‐L‐D‐Co_3_O_4_/CC cathode, which exhibits remarkable catalytic properties toward both OER and ORR. The outstanding OER and ORR performance of ZIF‐L‐D‐Co_3_O_4_/CC can be attributed to the unique hierarchical 3D‐on‐2D structure with multiple electrocatalytic activity sites and free‐standing architecture, which undoubtedly boosts reaction kinetics and mass transport of ionic species. Furthermore, the close protection of N‐doped carbon on Co_3_O_4_ nanoparticles, offers excellent electrical conductivity and electrochemical stability, resulting in enhanced OER and ORR stability. For the practical application, a flexible all‐solid‐state ZAB designed using the ZIF‐L‐D‐Co_3_O_4_/CC as an air cathode exhibits high open circuit potential (1.461 V), high capacity (815 mAh g^−1^ Zn at 1 mA cm^−2^), high energy density (1010 Wh kg^−1^ Zn), excellent cycling stability as well as outstanding mechanical flexibility. Particularly, as a flexible battery, the ZIF‐L‐D‐Co_3_O_4_/CC‐based all‐solid‐state ZAB can keep perfectly working even under different bending radius and angles conditions, indicating its successful application to realistic wearable electronic devices. It is believed that this work can push the enormous advance of next‐generation flexible energy conversion and storage devices.

## 4 Experimental Section

*Material Synthesis—Preparation of ZIF‐L Microarrays on Carbon Cloth (ZIF‐L/CC)*: Typically, 40 mL aqueous Co(NO_3_)_2_·6H_2_O (99%, 0.71 g) was quickly added into 40 mL aqueous 2‐methylimidazole (C_4_H_6_N_2_, 98%, 1.31 g) and then was stirred well. In a few minutes after that a piece of acid‐treated CC (2 × 4 × 0.036 cm^3^) was partly immersed into the mixture solution. After standing for 8 h at room temperature, the ZIF‐L/CC sample was taken out and cleaned with deionized water, followed by drying in a vacuum oven overnight. In addition, ZIF‐L‐1/CC, ZIF‐L‐2/CC, ZIF‐L‐3/CC, ZIF‐L‐4/CC, and ZIF‐L‐5/CC were prepared via the same procedure but with different amounts of Co(NO_3_)_2_·6H_2_O: 0.31 g for ZIF‐L‐1/CC, 0.41 g for ZIF‐L‐2/CC, 0.51 g for ZIF‐L‐3/CC, 0.61 g for ZIF‐L‐4/CC, and 0.81 g for ZIF‐L‐5/CC.

*Material Synthesis—Preparation of ZIF‐L‐D Nano‐Micro Arrays on Carbon Cloth (ZIF‐L‐D/CC)*: 0.71 g Co(NO_3_)_2_·6H_2_O and 1.61 g 2‐methylimidazole were separately dispersed in 40 mL methanol (99.5%) and then mixed under stirring. And then, the as‐prepared ZIF‐L/CC was further immersed into the mixture solution. After reaction for 2 h, the ZIF‐L‐D/CC sample was taken out, thoroughly cleaned with methanol and vacuum dried overnight. The mass loading of ZIF on CC was about 3.0 mg cm^−2^.

*Material Synthesis—Preparation of ZIF‐L‐D‐Co_3_O_4_ Nano‐Micro Arrays on Carbon Cloth (ZIF‐L‐D‐Co_3_O_4_/CC)*: The ZIF‐L‐D‐Co_3_O_4_/CC was obtained by means of a facile carbonization‐oxidation process. Briefly, a piece of ZIF‐L‐D arrays on carbon cloth was heated to 800 °C for 2 h with a ramp rate of 1 °C min^−1^ under Ar/N_2_ atmosphere, after cooling down to room temperature, the sample was transferred to muffle furnace and annealed in air at 300 °C for 60 min with a heating rate of 1 °C min^−1^. Finally, the mass loading of ZIF‐L‐D‐Co_3_O_4_/CC was about 2.1 mg cm^−2^.

*Material Synthesis—Preparation of ZIF‐L‐Co_3_O_4_ Microarrays on Carbon Cloth (ZIF‐L‐Co_3_O_4_/CC)*: Briefly, the as‐prepared ZIF‐L/CC was experienced the same carbonization‐oxidation process with that of the ZIF‐L‐D‐Co_3_O_4_/CC, and then the final product of ZIF‐L‐Co_3_O_4_/CC was obtained. The mass loading of ZIF‐L‐Co_3_O_4_/CC was about 1.2 mg cm^−2^.

*Material Synthesis—Preparation of ZIF‐D‐Co_3_O_4_ on Carbon Cloth (ZIF‐D‐Co_3_O_4_/CC)*: The ZIF‐D‐Co_3_O_4_/CC was obtained using the same procedure with that of the ZIF‐L‐D‐Co_3_O_4_/CC, but there was no need for the growth of ZIF‐L on CC. The mass loading of ZIF‐D‐Co_3_O_4_/CC was about 0.4 mg cm^−2^.

*Material Synthesis—Preparation of Pt/C/CC and IrO_2_/CC Electrode*: Commercial Pt/C (20 wt%, Alfa Aesar) or IrO_2_ (99.9%, Ir ≥ 84.5%, Aladdin) was well‐dispersed in 5% Nafion solution (10 µL diluted water, 30 µL ethanol, and 60 µL Nafion) under ultrasonic treatment at room temperature for 30 min. Then, the suspension was coated to CC with similar mass loading to that of the ZIF‐L‐D‐Co_3_O_4_/CC. The final samples were naturally dried before test.

*Material Characterization*: The crystal configuration and crystallographic plane of synthetic materials were identified by XRD (Ultima IV Germany). The specific surface area and the pore diameter distribution were tested at liquid nitrogen temperature (77 K) by surface area and porosimetry analyzer (V‐Sorb 2800P). The SEM (JEOL JSM‐6380LA) and TEM (JEOL JEM‐2100HR) were carried out to observe the morphology, structure as well as particle size of the samples. XPS (Thermo Fisher Scientific, UK) was used with monochromatic Al‐Ka X‐ray source (excitation energy = 1468.6 eV) under ultrahigh vacuum (lower than 5 × 10^−8^ mbar). Spectra were collected from 0 to 1350 eV using an X‐ray spot size of 400 µm with pass energy 100 eV for wide scan and 30 eV for individual elements. Binding energies were corrected based on the carbon 1s signal at 284.8 eV.

*Electrochemical Measurements*: a) The OER and ORR performances were marked by electrochemical workstation CHI760E (Shanghai Chenhua, China) with a sample‐modified glassy carbon as working electrode (the sample was immobilized on the glassy carbon rotating disc electrode tip, *S* = 0.196 cm^2^), a Pt foil, and an Ag/AgCl as the counter and reference electrodes respectively, establishing a standard three‐electrode system in 1 m KOH electrolyte in an oxygenated environment. The scan rate for OER and ORR measurements were 5 mV s^−1^ and for their accelerated durability tests were 50 mV s^−1^. And the electrochemical activity of catalyst was studied via linear sweeping voltammetry on GAMRY RDE710 rotating electrode at various rotating speeds ranging from 400 to 2400 rpm. All measured potentials were calibrated to reversible hydrogen electrode (RHE, *E*_RHE_ = *E*_Ag/AgCl(saturated KCl)_ + 0.0592 × pH + 0.1972). OER and ORR time‐dependent stability were represented by the chronoamperometric response at a constant current density and chronopotentiometric responses at a constant overpotential. And the number of electron transferred (*n*) during ORR, however, was calculated from slope of Koutecky–Levich (K–L) plots on the basis of the K–L equation

(1) $$\frac{1}{i}=\frac{1}{i_{k}}+\frac{1}{i_{\mathrm{dl}}}=\frac{1}{i_{k}}+\frac{1}{0.62nFAC_{O_{2}}D_{O_{2}}^{2/3}v^{-1/6}\omega^{1/2}}$$

(2) $$\mathrm{slope}={\left(0.62nFAC_{O_{2}}D_{O_{2}}^{2/3}v^{-1/6}\right)}^{-1}$$

where *F* is Faraday constant (96 500 C mol^−1^), *A* is the area of glassy carbon electrode (*S* = 0.196 cm^−2^), and ν is the kinetic viscosity of the electrolyte (9.5 × 10^−3^ cm^−2^ s^−1^). *C* ${}_{O_{2}}$ is the concentration of O_2_ in 1 m KOH solution at 25 °C and partial pressure of 1 atm (8.3 × 10^−7^ mol cm^−3^), while *D* ${}_{O_{2}}$ is the diffusion coefficient of O_2_ under the same condition (1.65 × 10^−5^ cm^−2^ s^−1^).

b) The aqueous ZABs were assembled using ZIF‐L‐D‐Co_3_O_4_/CC attached to the water‐facing side of hydrophobic carbon cloth (W1S1005, include micro porous layer) as the air cathode, a polished Zn foil as the anode, and the mixed aqueous solution of 6 m KOH and 0.1 m zinc acetate as the electrolyte, with the exposed area of the air cathode (to the electrolyte and air) to be ≈1 cm^2^. And then, the assembled aqueous ZABs were patiently tested on a multichannel battery tester (Land CT2001A, Wuhan, China) at room temperature, on which cycling test was conducted via charge–discharge cycles at constant current density for 10 min of discharge and 10 min of charge at 5 mA cm^−2^. Polarization curves were recorded by electrochemical workstation CHI760E (Shanghai Chenhua, China) via linear sweeping voltammetry at a scan rate of 5 mV s^−1^. To obtain the full discharge profiles of batteries, the change in voltage was recorded at a current density of 5 mA cm^−2^, while the specific capacity was calculated in accordance with the mass of Zn anode in assembled batteries.

c) To assemble the flexible solid‐state ZAB, a PVA‐based gel polymer was employed as the solid electrolyte. Briefly, 10 g PVA (MW = 190 000, Aladdin) powder was poured into 80 mL distilled water at 90 °C under stirring until the solution became transparent and homogeneous. And then 20 mL of 9 m KOH was added dropwise under stirring for another 60 min at 80 °C. Subsequently, a filter paper, fully soaked with the gel electrolyte, was used as separator sandwiched between a polished Zn foil (2 × 2 cm^2^, with another 2 × 2 cm^2^ left blank as current collector) and the sample electrode (2 × 2 cm^2^, with another 2 × 2 cm^2^ left blank as current collector). Lastly during assembly, the white breathable tapes (Scotch, 3M) were utilized for sealing the device, and the final assembled solid‐state ZABs were about 0.3 cm in thickness. Cycling test was conducted on battery testers (Land CT2001A, Wuhan, China) via charge–discharge cycles at constant current density for 10 min of discharge and 10 min of charge at 1 mA cm^−2^. Moreover, mechanical stability and flexibility tests were implemented with altered state between flat and fold for every three cycles. Polarization curves were recorded at a scan rate of 5 mV s^−1^ and full discharge profiles were collected at a current density of 1 mA cm^−2^. The electrochemical impedance spectroscopy of battery was carried out on an Autolab (PGSTAT302N) with AC signal 10 mV rms from 0.1 MHz to 0.01 Hz.

*Disclaimer*: The volunteer involving in all demonstration experiments was the first author of this manuscript, Y.T. Zhong, who consented to the experiments and to the presentation of his demonstrations in this manuscript.

## Conflict of Interest

The authors declare no conflict of interest.

## Supporting information

Supplementary

Supplementary

## References

[1] C. Guan , A. Sumboja , W. Zang , Y. Qian , H. Zhang , X. Liu , Z. Liu , D. Zhao , S. J. Pennycook , J. Wang , Energy Storage Mater. 2019, 16, 243.

[2] L. An , Y. Li , M. Luo , J. Yin , Y.‐Q. Zhao , C. Xu , F. Cheng , Y. Yang , P. Xi , S. Guo , Adv. Funct. Mater. 2017, 27, 1703779.

[3] H. Yang , B. Wang , H. Li , B. Ni , K. Wang , Q. Zhang , X. Wang , Adv. Energy Mater. 2018, 8, 1801839.

[4] L. Yang , L. Shi , D. Wang , Y. Lv , D. Cao , Nano Energy 2018, 50, 691.

[5] Z. Zhao , Z. Yuan , Z. Fang , J. Jian , J. Li , M. Yang , C. Mo , Y. Zhang , X. Hu , P. Li , S. Wang , W. Hong , Z. Zheng , G. Ouyang , X. Chen , D. Yu , Adv. Sci. 2018, 5, 1800760. 10.1002/advs.201800760 PMC6299824 30581696

[6] X. Han , G. He , Y. He , J. Zhang , X. Zheng , L. Li , C. Zhong , W. Hu , Y. Deng , T.‐Y. Ma , Adv. Energy Mater. 2018, 8, 1702222.

[7] Z. Pei , Z. Tang , Z. Liu , Y. Huang , Y. Wang , H. Li , Q. Xue , M. Zhu , D. Tang , C. Zhi , J. Mater. Chem. A 2018, 6, 489.

[8] Y. Huang , Z. Li , Z. Pei , Z. Liu , H. Li , M. Zhu , J. Fan , Q. Dai , M. Zhang , L. Dai , C. Zhi , Adv. Energy Mater. 2018, 8, 1802288.

[9] C. Guan , A. Sumboja , H. Wu , W. Ren , X. Liu , H. Zhang , Z. Liu , C. Cheng , S. J. Pennycook , J. Wang , Adv. Mater. 2017, 29, 1704117. 10.1002/adma.201704117 29024075

[10] J. Fu , F. M. Hassan , J. Li , D. U. Lee , A. R. Ghannoum , G. Lui , M. A. Hoque , Z. Chen , Adv. Mater. 2016, 28, 6421. 27197721 10.1002/adma.201600762

[11] C. Guan , W. Zhao , Y. Hu , Z. Lai , X. Li , S. Sun , H. Zhang , A. K. Cheetham , J. Wang , Nanoscale Horiz. 2017, 2, 99. 10.1039/c6nh00224b 32260671

[12] Q. Zhang , Z. Zhou , Z. Pan , J. Sun , B. He , Q. Li , T. Zhang , J. Zhao , L. Tang , Z. Zhang , L. Wei , Y. Yao , Adv. Sci. 2018, 5, 1801462. 10.1002/advs.201801462 PMC6299715 30581717

[13] A. Sumboja , M. Lübke , Y. Wang , T. An , Y. Zong , Z. Liu , Adv. Energy Mater. 2017, 7, 1700927.

[14] P. Tan , B. Chen , H. Xu , W. Cai , W. He , M. Liu , Z. Shao , M. Ni , Small 2018, 14, 1800225. 10.1002/smll.201800225 29682867

[15] J. Pan , Y. Y. Xu , H. Yang , Z. Dong , H. Liu , B. Y. Xia , Adv. Sci. 2018, 5, 1700691. 10.1002/advs.201700691 PMC5908379 29721418

[16] H.‐F. Wang , C. Tang , Q. Zhang , Adv. Funct. Mater. 2018, 28, 1803329.

[17] H Zhang , T. Wang , A. Sumboja , W. Zang , J. Xie , D. Gao , S. J. Pennycook , Z. Liu , C. Guan , J. Wang , Adv. Funct. Mater. 2018, 28, 1804846.

[18] J. S. Jang , W. T. Koo , D. H. Kim , I. D. Kim , ACS Cent. Sci. 2018, 4, 929. 30062121 10.1021/acscentsci.8b00359 PMC6062837

[19] J. Zhang , T. Zhang , K. Xiao , S. Cheng , G. Qian , Y. Wang , Y. Feng , Cryst. Growth Des. 2016, 16, 6494.

[20] X. Liu , C. Guan , Y. Hu , L. Zhang , A. M. Elshahawy , J. Wang , Small 2018, 14, 1702641. 10.1002/smll.201702641 29076649

[21] R. Chen , J. Yao , Q. Gu , S. Smeets , C. Baerlocher , H. Gu , D. Zhu , W. Morris , O. M. Yaghi , H. Wang , Chem. Commun. 2013, 49, 9500. 10.1039/c3cc44342f 24018656

[22] S. Gadipelli , T. Zhao , S. A. Shevlin , Z. Guo , Energy Environ. Sci. 2016, 9, 1661.

[23] T. Sun , J. Wang , C. Qiu , X. Ling , B. Tian , W. Chen , C. Su , Adv. Sci. 2018, 5, 1800036. 10.1002/advs.201800036 PMC6051395 30027038

[24] F. Shi , Z. Geng , K. Huang , Q. Liang , Y. Zhang , Y. Sun , J. Cao , S. Feng , Adv. Sci. 2018, 5, 1800575. 10.1002/advs.201800575 PMC6096989 30128261

[25] H.‐F. Wang , C. Tang , B. Wang , B.‐Q. Li , X. Cui , Q. Zhang , Energy Storage Mater. 2018, 15, 124.

[26] M chen , Y Zhang , L Xing , Y Liao , Y Qiu , S Yang , W. Li , Adv. Mater. 2017, 29, 1607015. 10.1002/adma.201607015 28558122

[27] I. S. Amiinu , X. Liu , Z. Pu , W. Li , Q. Li , J. Zhang , H. Tang , H. Zhang , S. Mu , Adv. Funct. Mater. 2018, 28, 1704638.

[28] J. Wang , W. Cui , Q. Liu , Z. Xing , A. M. Asiri , X. Sun , Adv. Mater. 2016, 28, 215. 26551487 10.1002/adma.201502696

[29] Q. Liu , Y. Wang , L. Dai , J. Yao , Adv. Mater. 2016, 28, 3000. 26914270 10.1002/adma.201506112

[30] S. Gadipelli , Z. Li , T. Zhao , Y. Yang , T. Yildirim , Z. Guo , J. Mater. Chem. A 2017, 5, 24686.

[31] A. P. Tiwari , D. Kim , Y. Kim , H. Lee , Adv. Energy Mater. 2017, 7, 1602217.

[32] Q. Ren , H. Wang , X. Lu , Y. Tong , G. Li , Adv. Sci. 2018, 5, 1700515. 10.1002/advs.201700515 PMC5867057 29593954

[33] T. Wang , Z. Kou , S. Mu , J. Liu , D. He , I. S. Amiinu , W. Meng , K. Zhou , Z. Luo , S. Chaemchuen , F. Verpoort , Adv. Funct. Mater. 2018, 28, 1705048.

[34] Y. Zhong , B. Li , S. Li , S. Xu , Z. Pan , Q. Huang , L. Xing , C. Wang , W. Li , Nano‐Micro Lett. 2018, 10, 56. 10.1007/s40820-018-0209-1 PMC6199101 30393704

[35] L. Zhu , D. Zheng , Z. Wang , X. Zheng , P. Fang , J. Zhu , M. Yu , Y. Tong , X. Lu , Adv. Mater. 2018, 30, 1805268. 10.1002/adma.201805268 30259586

[36] W. Qiu , Y. Li , A. You , Z. Zhang , G. Li , X. Lu , Y. Tong , J. Mater. Chem. A 2017, 5, 14838.

[37] J. Yin , Y. Li , F. Lv , M. Lu , K. Sun , W. Wang , L. Wang , F. Cheng , Y. Li , P. Xi , S. Guo , Adv. Mater. 2017, 29, 1704681. 10.1002/adma.201704681 29239518

[38] I. S. Amiinu , Z. Pu , X. Liu , K. A. Owusu , H. G. R. Monestel , F. O. Boakye , H. Zhang , S. Mu , Adv. Funct. Mater. 2017, 27, 1702300.

[39] B. Liao , H. Li , M. Xu , L. Xing , Y. Liao , X. Ren , W. Fan , L. Yu , K. Xu , W. Li , Adv. Energy Mater. 2018, 8, 1800802.

[40] M. Chen , D. Chen , Y. Liao , X. Zhong , W. Li , Y. Zhang , ACS Appl. Mater. Interfaces 2016, 8, 4575. 26799282 10.1021/acsami.5b10219

[41] X. Q. Chen , H. B. Lin , X. W. Zheng , X. Cai , P. Xia , Y. M. Zhu , X. P. Li , W. S. Li , J. Mater. Chem. A 2015, 3, 18198.

[42] H.‐F. Wang , C. Tang , B. Wang , B.‐Q. Li , Q. Zhang , Adv. Mater. 2017, 29, 1702327.

[43] W. Ren , H. Zhang , C. Guan , C. Cheng , Adv. Funct. Mater. 2017, 27, 1702116.

[44] K. Jayaramulu , J. Masa , D. M. Morales , O. Tomanec , V. Ranc , M. Petr , P. Wilde , Y.‐T. Chen , R. Zboril , W. Schuhmann , R. A. Fischer , Adv. Sci. 2018, 5, 1801029. 10.1002/advs.201801029 PMC6247023 30479932

[45] L. Ma , S. Chen , Z. Pei , H. Li , Z. Wang , Z. Liu , Z. Tang , J. A. Zapien , C. Zhi , ACS Nano 2018, 12, 8597. 30040383 10.1021/acsnano.8b04317

[46] Z. Pei , Y. Huang , Z. Tang , L. Ma , Z. Liu , Q. Xue , Z. Wang , H. Li , Y. Chen , C. Zhi , Energy Storage Mater. 2018, 10.1016/j.ensm.2018.11.010.

