# Supplementary material for: Hierarchical Co3O4 Nano‐Micro Arrays Featuring Superior Activity as Cathode in a Flexible and Rechargeable Zinc–Air Battery
Source: Adv Sci (Weinh). 2019 Mar 26;6(11):1802243. doi: 10.1002/advs.201802243 (PMC6548986; doi:10.1002/advs.201802243)
Supplement: Supplementary file 1 — Supplementary [file ADVS-6-1802243-s002.pdf]

Copyright WILEY-VCH Verlag GmbH & Co. KGaA, 69469 Weinheim, Germany, 2019.

ADVANCED SCIENCE
Open Access

Supporting Information

for *Adv. Sci.,* DOI: 10.1002/advs.201802243

Hierarchical Co_3O_4 Nano-Micro Arrays Featuring Superior Activity as Cathode in a Flexible and Rechargeable Zinc–Air Battery

Yaotang Zhong, Zhenghui Pan, Xianshu Wang, Jie Yang, Yongcai Qiu, Shuyuan Xu, Yitong Lu, Qiming Huang,* and Weishan Li**

Supporting Information

Hierarchical Co_3O_4 Nano-micro Arrays Featuring Superior Activity as Cathode in Flexible and Rechargeable Zinc-Air Battery

Yaotang Zhong[a], Zhenghui Pan[a,b*], Xianshu Wang,[a] Jie Yang,[c] Yongcai qiu[c], Shuyuan Xu[a], Yitong Lu[a], Qiming Huang[a,d*], Weishan Li[a,d*]

[a]School of Chemistry and Environment, South China Normal University, Guangzhou 510006, China

[b]Department of Materials Science and Engineering, National University of Singapore, 117574 Singapore, Singapore

[c]School of Environment & Energy, South China University of Technology, Guangzhou 510006, Guangdong, China

[d]Engineering Research Center of MTEES (Ministry of Education), Research Center of BMET (Guangdong Province), and Key Laboratory of ETESPG (GHEI), South China Normal University, Guangzhou 510006, China

Email address: liwsh@scnu.edu.cn; msepz@nus.edu.sg; huangqm@scnu.edu.cn

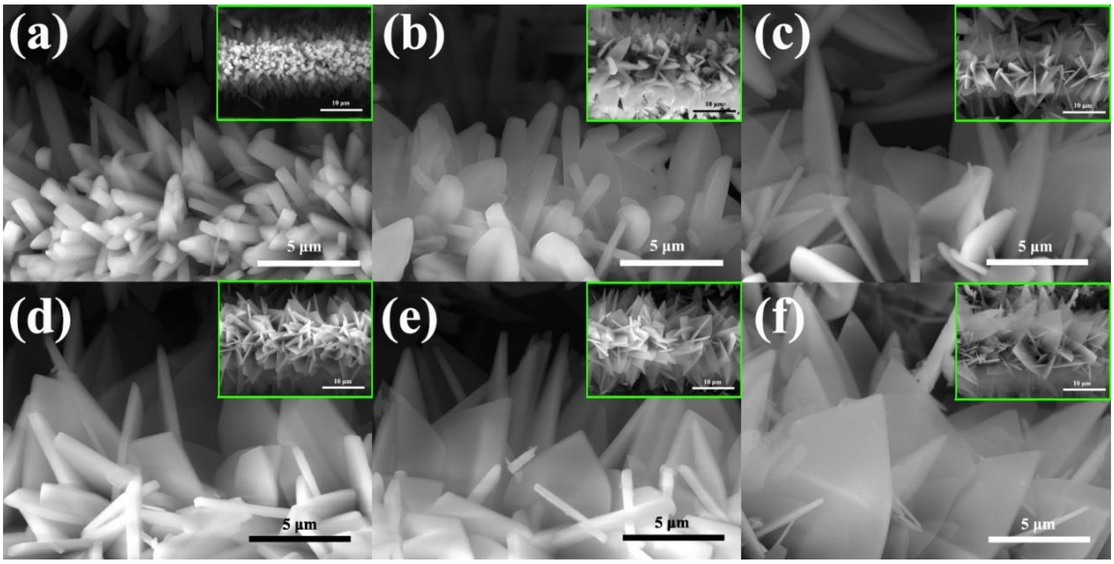

Figure S1. SEM images of (a) ZIF-L-1/CC, (b) ZIF-L-2/CC, (c) ZIF-L-3/CC, (d) ZIF-L-4/CC, (e) ZIF-L/CC, and (f) ZIF-L-5/CC.

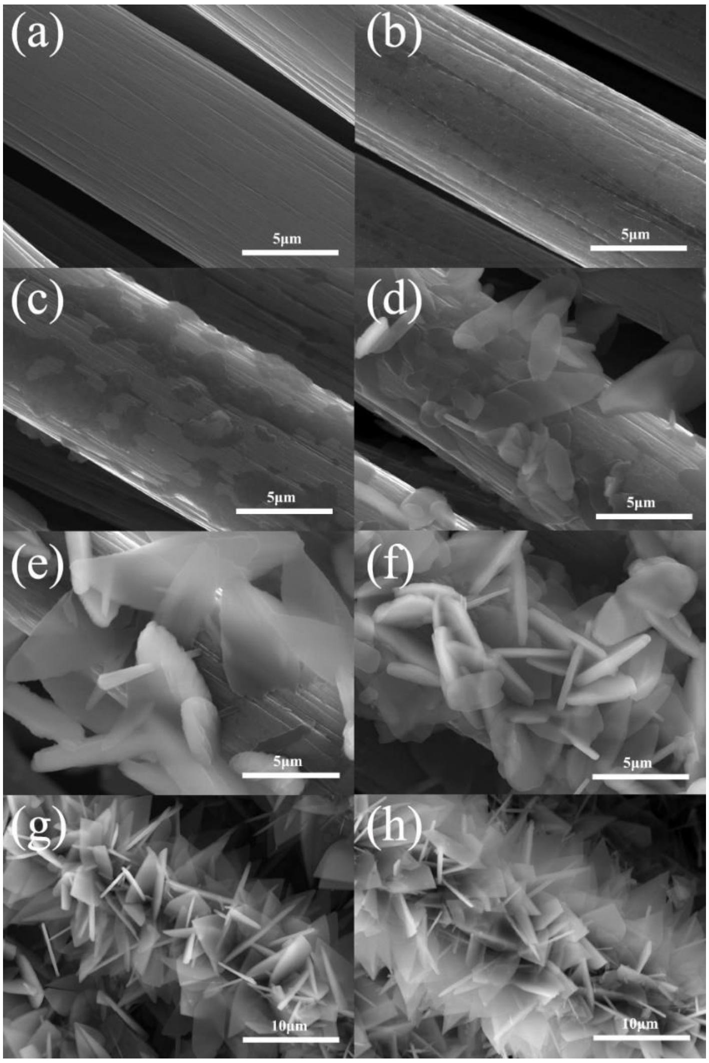

Figure S2. SEM images of ZIF-L/CC at different growth time, (a) 0 min, (b) 15 min, (c) 30 min, (d) 1 h, (e) 2 h, (f) 4 h, (g) 8 h, and (h) 12 h.

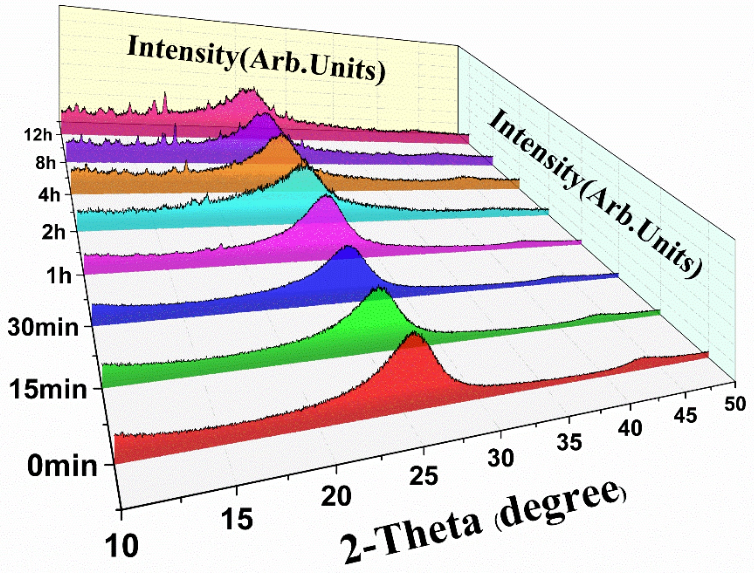

Figure S3. Evolution in XRD patterns of ZIF-L/CC with growth time.

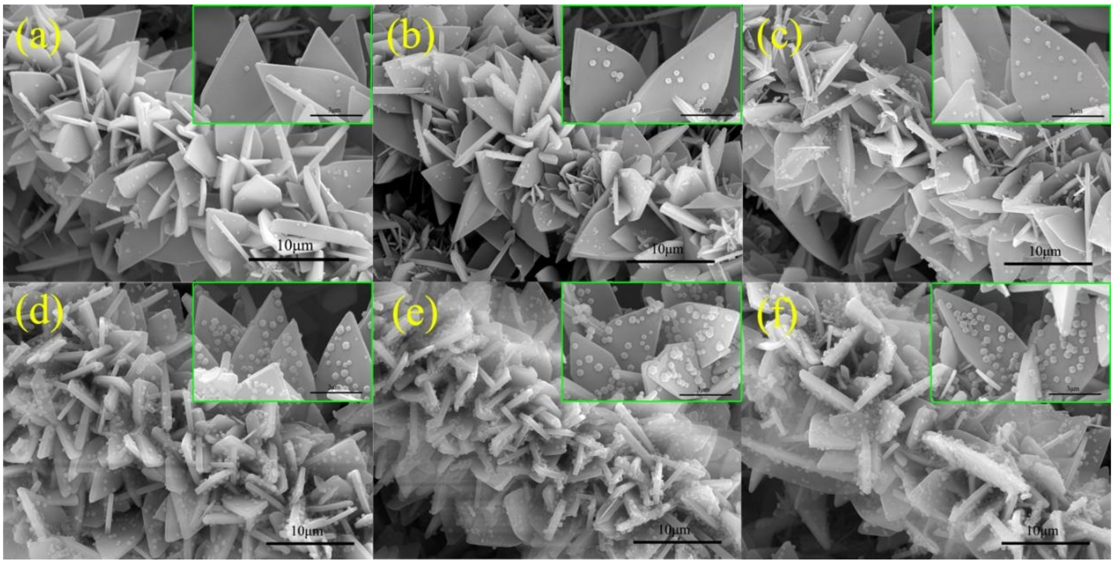

Figure S4. SEM images of ZIF-L-D/CC at different growth time, (a) 5 min, (b) 15 min, (c) 30 min, (d) 1 h, (e) 2 h, and (f) 4 h.

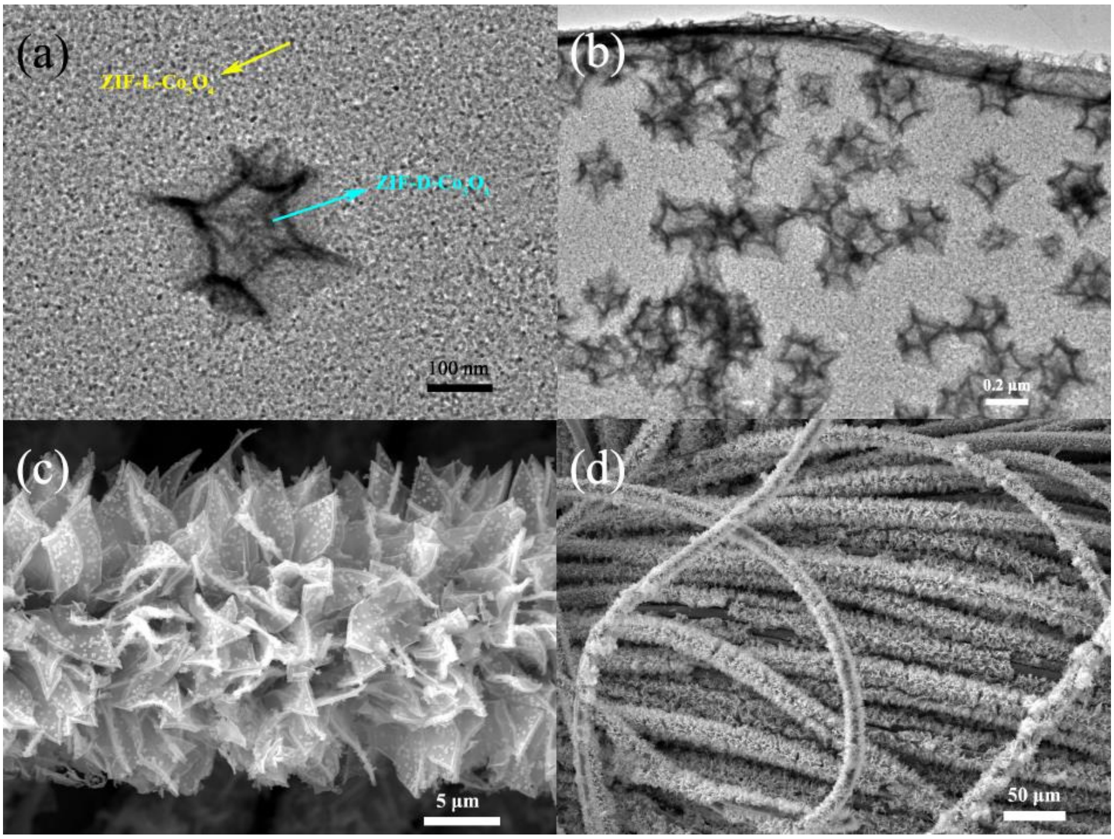

Figure S5. (a, b) TEM and (c, d) SEM images of ZIF-L-D-Co_3O_4/CC.

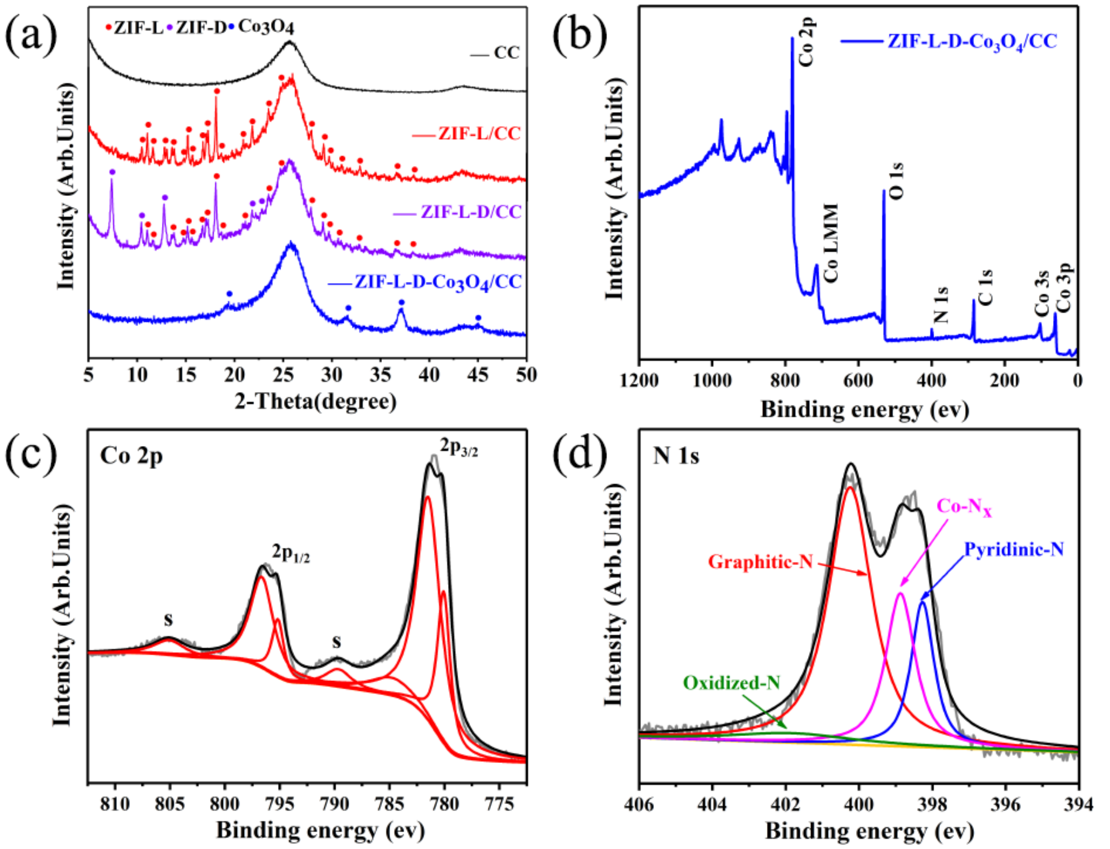

Figure S6. (a) XRD patterns of precursors and ZIF-L-D-Co_3O_4/CC. (b) XPS profile, (c) Co 2p and (d) N 1s spectra of ZIF-L-D-Co_3O_4/CC.

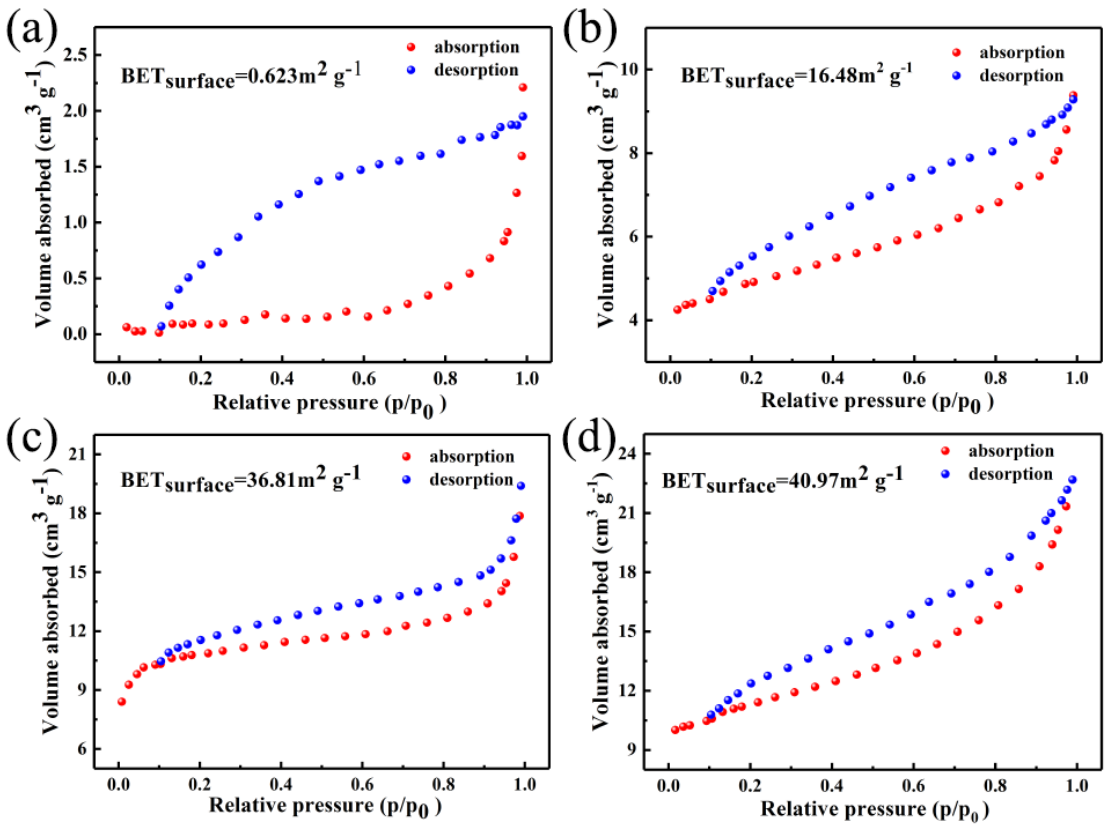

Figure S7. Adsorption/desorption profiles of (a) CC, (b) ZIF-L/CC, (c) ZIF-L-D/CC, and (d) ZIF-L-D-Co_3O_4/CC.

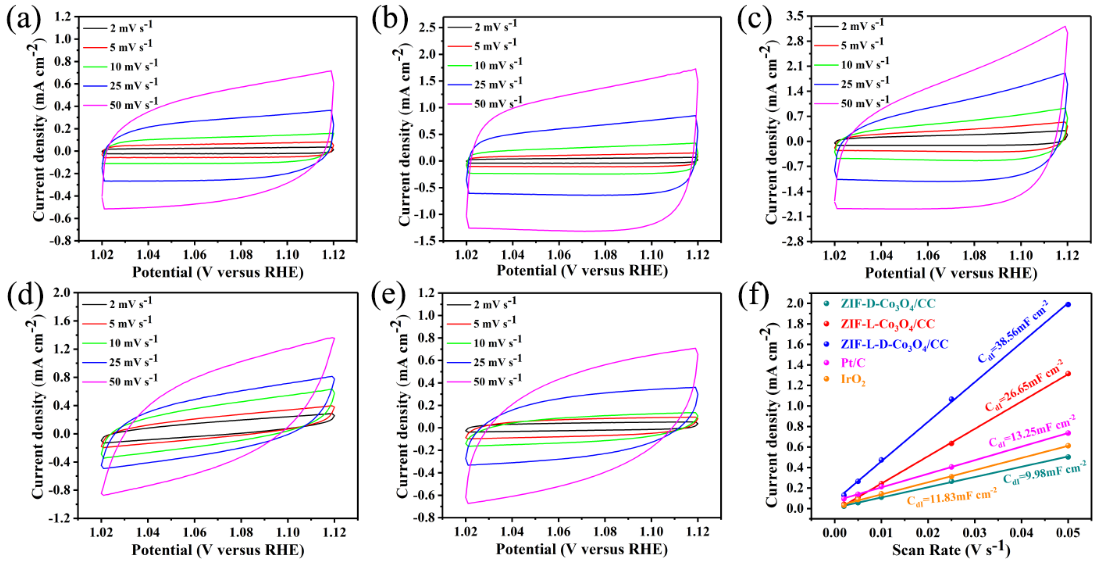

Figure S8. (a-e) The CVs collected at different scanning rates of (a) ZIF-D-Co_3O_4/CC, (b) ZIF-L-Co_3O_4/CC, (c) ZIF-L-D-Co_3O_4/CC, (d) Pt/C, and (e) IrO_2. (f) Relations of the current density and the scanning rate.

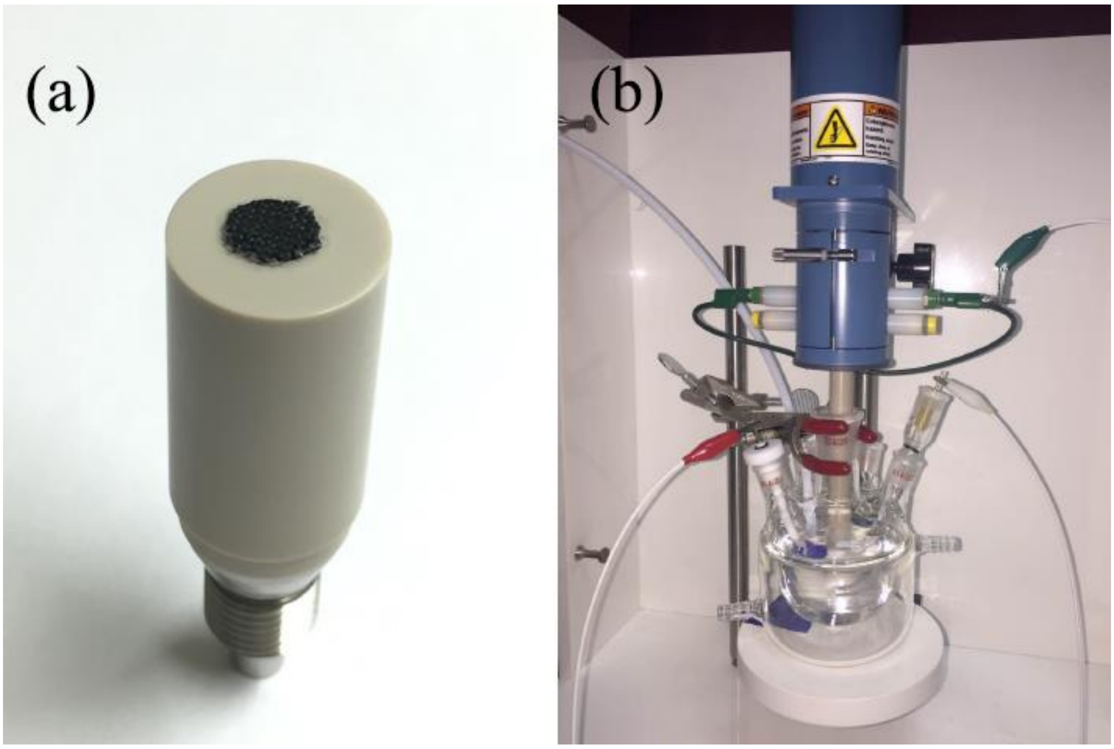

Figure S9. Digital images showing (a) samples on CC directly attached to glassy carbon electrode and (b) the test device of rotating disk electrode.

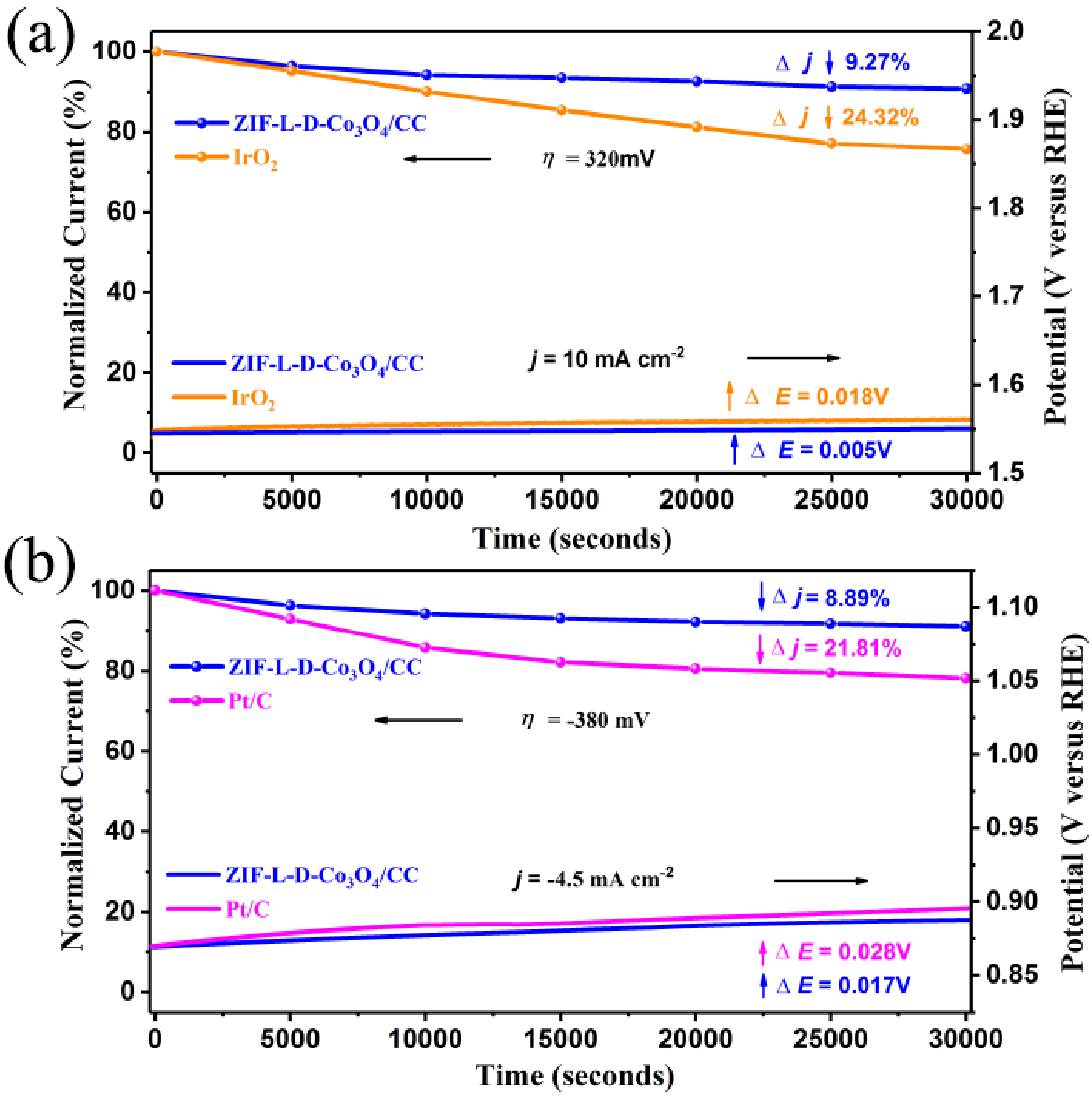

Figure S10. Chronoamperometric responses at a constant current density and chronopotentiometric responses at a constant overpotential for (a) OER and (b) ORR.

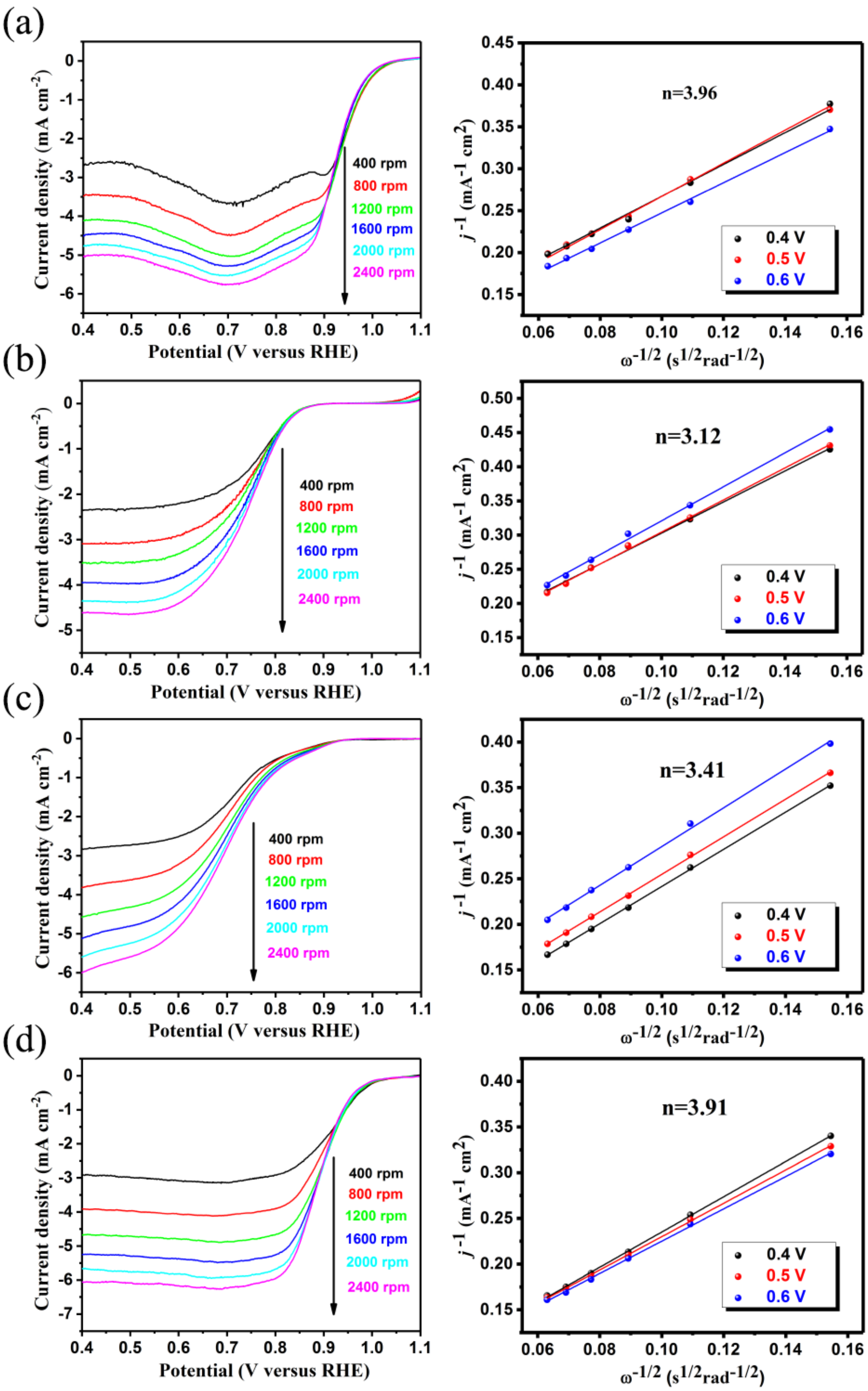

Figure S11. ORR polarization curves recorded at different rotation speeds with the corresponding Koutechky–Levich (K-L) plots of (a) Pt/C, (b) ZIF-D-Co_3O_4/CC, (c) ZIF-L-Co_3O_4/CC, and (d) ZIF-L-D-Co_3O_4/CC.

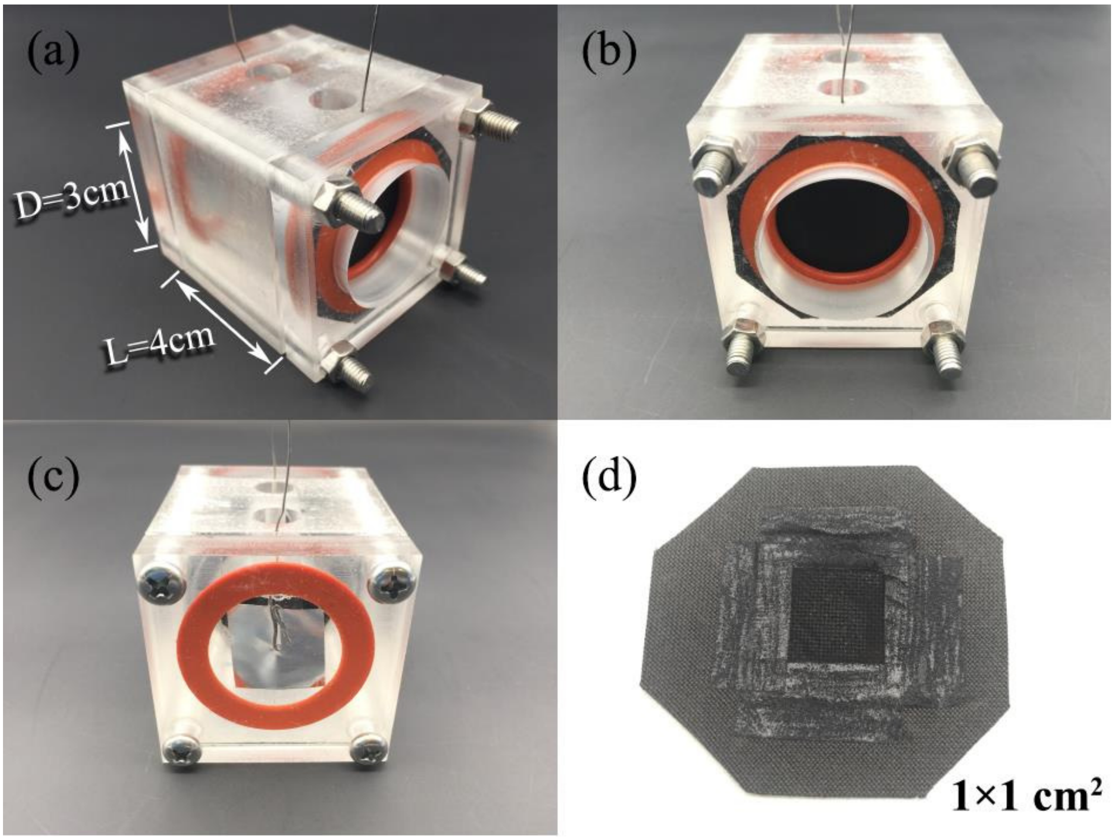

Figure S12. Digital images of (a) the assembled aqueous ZAB, (b) the air cathode side, (c) the Zn foil side of aqueous ZABs, and (d) the sample on CC (the exposed area is about 1 cm^{-2}) directly attached to water-facing side of hydrophobic CC using conducting tapes.

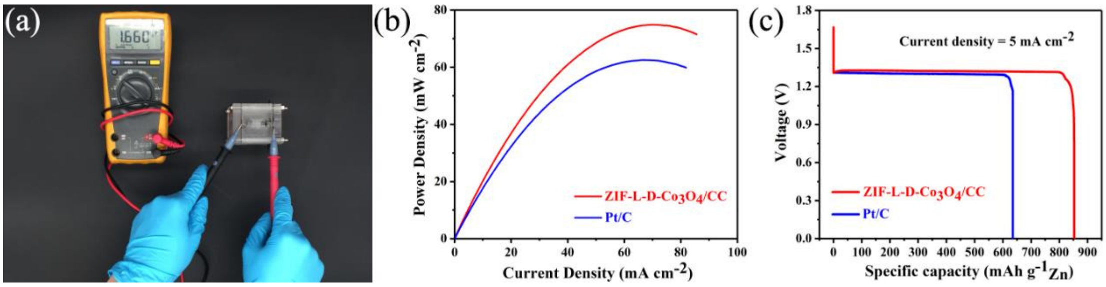

Figure S13. (a) Photograph of the ZIF-L-D-Co_3O_4/CC-based aqueous ZAB exhibiting an open circuit voltage of 1.660 V. (b) Power–current density curves and (c) voltage–capacity curves of aqueous ZABs with air cathodes of ZIF-L-D-Co_3O_4/CC and Pt/C.

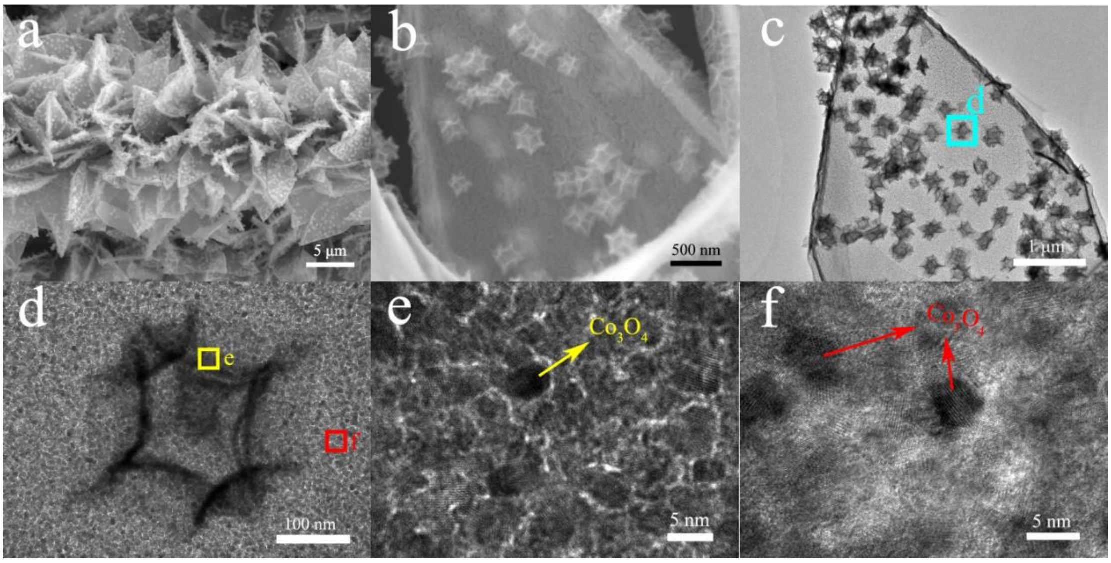

Figure S14. (a, b) SEM and (c, d) TEM images of ZIF-L-D-Co_3O_4/CC; HRTEM images of (e) ZIF-D-Co_3O_4 and (f) ZIF-L-Co_3O_4 after cycling test.

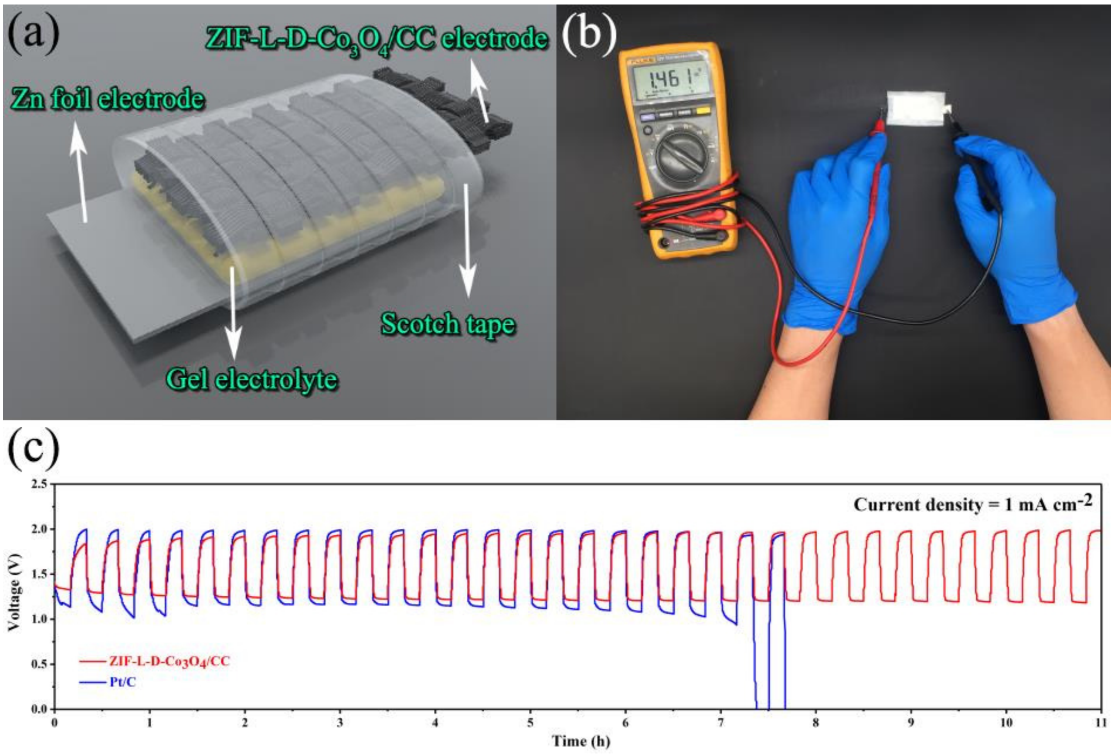

Figure S15. (a) Schematic representation and (b) photograph of the flexible ZIF-L-D-Co_3O_4/CC-based all-solid-state ZAB exhibiting an open circuit voltage of ≈1.461 V. (c) Comparison of stability between ZIF-L-D-Co_3O_4/CC and Pt/C as the air cathode in all-solid-state ZABs.

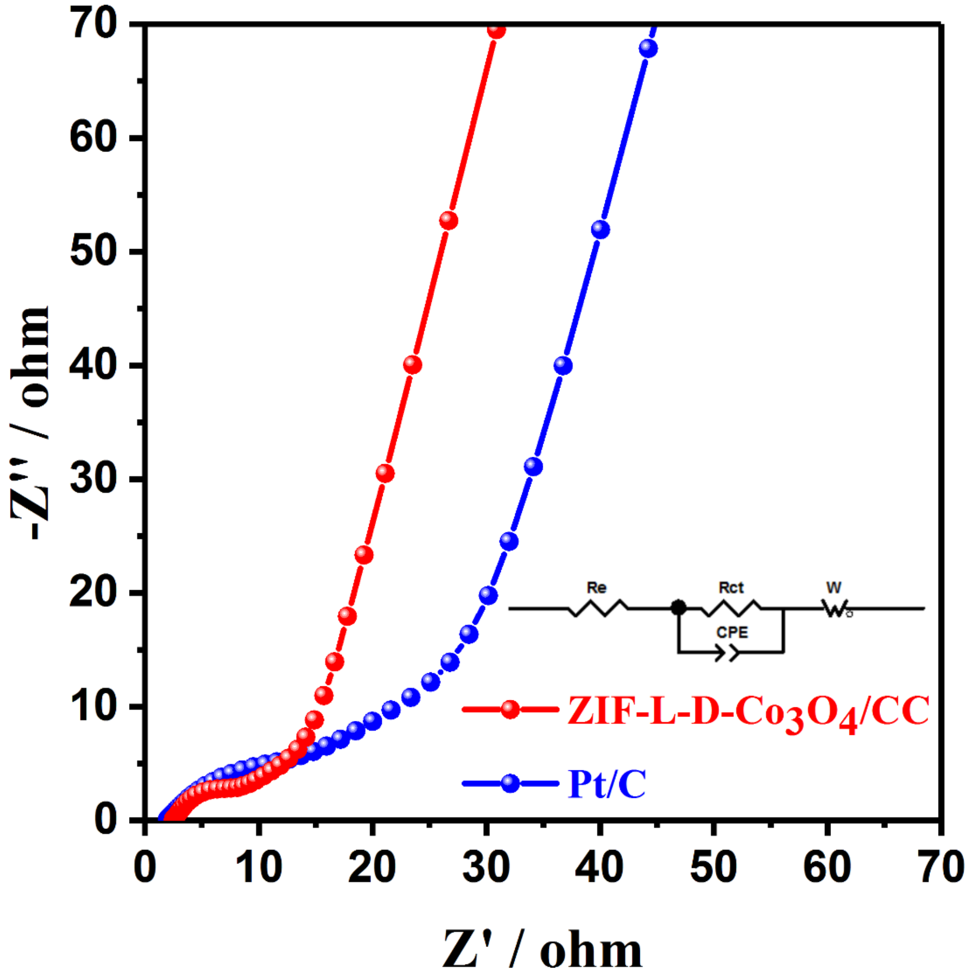

Figure S16. Electrochemical impedance spectra of the all-solid-state ZABs.

Table S1. Element contents in ZIF-L-D-Co_3O_4, obtained from XPS analyses

Element	Co (at%)	C (at%)	O (at%)	N (at%)
Content	27.93	24.29	40.12	7.66

Table S2. The mass loading of Co_3O_4 in different structural units of ZIF-L-D-Co_3O_4/CC

**Structural unit**	**Mass loading of Co_3O_4 (mg cm^{-2})**
3D-on-2D	1.82
3D	0.81
2D	1.01

Table S3. A comparison of our work with those recently reported in literature

**Catalysts**	**Loading density ($mg\ cm^{-2}$)**	**Current density ($mA\ cm^{-2}$)**	**Open circuit potential (V)**	**Voltage gap (V)**	**Reference**
NC-Co_3O_4	1.2	1	1.44	0.88	[9]
Co_3O_{4-x}	1.1	2	1.40	0.71	[45]
Co-NCNT	1.0	2	1.45	0.82	[46]
CoNC@Al_2O_3	-	20	1.42	0.90	[35]
Co_3O_4-NCNT/SS	1.1	25	1.42	0.80	[10]
NiS_2/CoS_2-O NWs	-	3	1.49	1.15	[37]
CuS/NiS_2 INs	2.0	25	1.41	0.92	[2]
ZIF-L-D-Co_3O_4/CC	2.1	1	1.46	0.83	This work
